# Visualizing incompatibilities in phylogenetic trees using consensus outlines

**DOI:** 10.3389/fbinf.2023.1155286

**Published:** 2023-06-01

**Authors:** Daniel H. Huson, Banu Cetinkaya

**Affiliations:** ^1^ Algorithms in Bioinformatics, University of Tübingen, Tübingen, Germany; ^2^ International Max Planck Research School “From Molecules to Organisms”, Max Planck Institute for Biology, Tübingen, Germany

**Keywords:** phylogenetics, consensus methods, phylogenetic network, visualization, software

## Abstract

Phylogenetic analysis frequently leads to the creation of many phylogenetic trees, either from using multiple genes or methods, or through bootstrapping or Bayesian analysis. A consensus tree is often used to summarize what the trees have in common. Consensus networks were introduced to also allow the visualization of the main incompatibilities among the trees. However, in practice, such networks often contain a large number of nodes and edges, and can be non-planar, making them difficult to interpret. Here, we introduce the new concept of a phylogenetic consensus outline, which provides a planar visualization of incompatibilities in the input trees, without the complexities of a consensus network. Furthermore, we present an effective algorithm for its computation. We demonstrate its usage and explore how it compares to other methods on a Bayesian phylogenetic analysis of languages using data from a published database and on multiple gene trees from a published study on water lilies.

## 1 Introduction

A main goal of phylogenetic analysis is to determine the evolutionary relationships and history of a set organisms. Often, the underlying data are DNA or protein sequences, and usually, projects give rise to many different phylogenetic trees, either because the analysis is performed individually on several different genes (Bapteste et al., 2002), or because different types of algorithms are applied to the same data, or because bootstrapping has been applied (Efron et al., 1996), or because a Bayesian analysis has produced a long chain of trees (Yang and Rannala, 1997).

For example, Rokas et al., 2003 use both maximum parsimony and maximum-likelihood (ML) methods to investigate the phylogenetic relationships between different yeast species, based on 106 genes. In (Leache and Reeder, 2002), multiple tree-inference methods, bootstrapping and consensus calculation are used to obtain a more robust identification of species among different lineages of the Eastern Fence Lizard. In the realm of human languages, Greenhill (2015) uses Bayesian phylogenetic analysis, involving the calculation of a profile of 10,000 trees, to determine the relationships between languages used on the Huon Peninsula. Gruenstaeudl (2019) uses a multi-gene approach, involving 78 different protein-coding genes, to investigate the phylogenetic relationships between water lilies and other aquatic plants.

In another paper, Wu et al., 2020 analyse 16 species of the genus *Paeonia*, using the complete chloroplast genome and also by considering different regions of the genome. They construct both maximum-likelihood and Bayesian trees. While application of the two methods to some regions leads to similar tree topologies, application to the inverted repeat (IR) region gives rise to incompatible trees. Moreover, maximum-likelihood analysis (with bootstrapping) on 19 highly variable regions of the chloroplast genome gives rise to several different topologies. Differences in topology are described in the paper in words.

**FIGURE 1:**
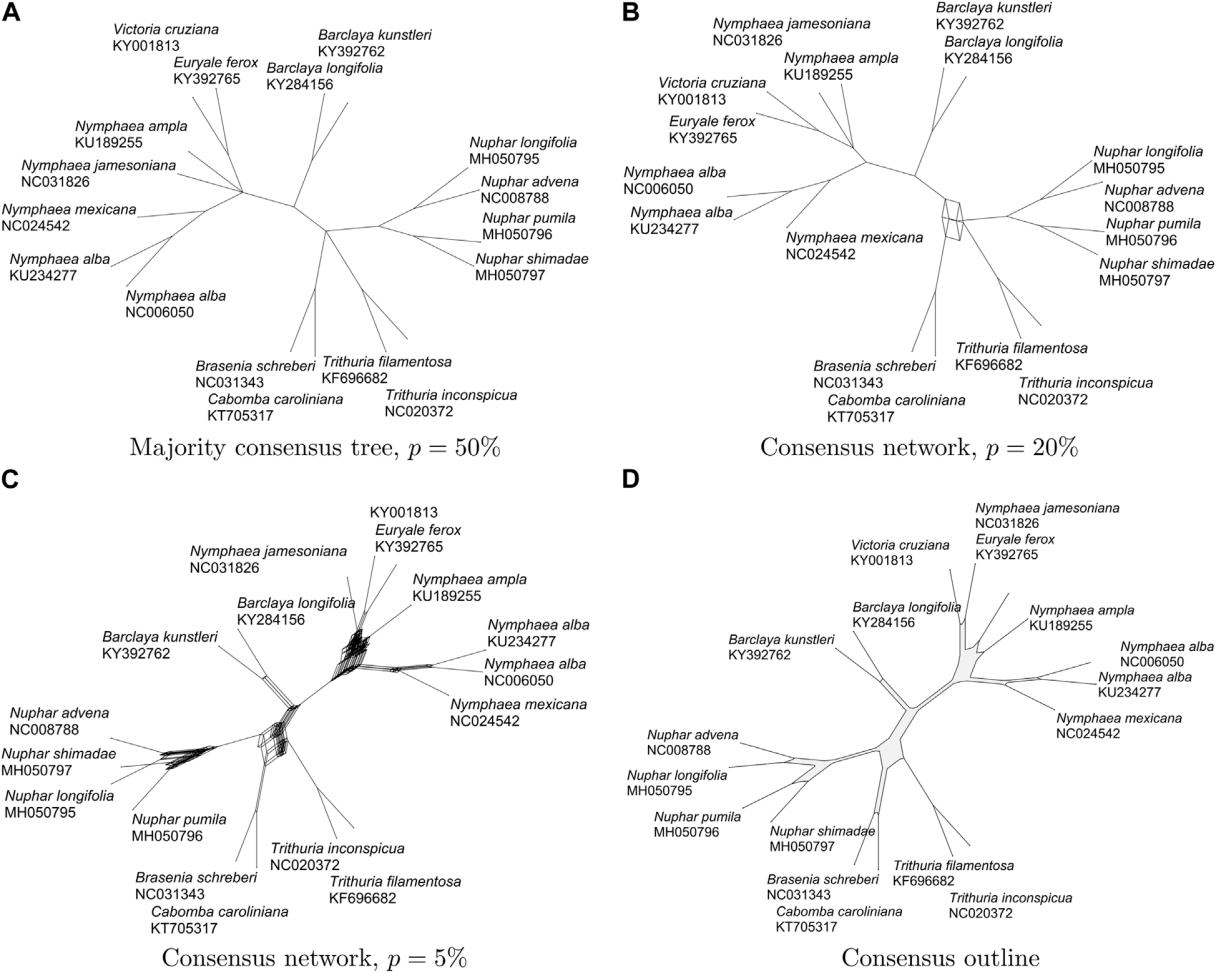
For 78 gene trees on 17 aquatic taxa [Gruenstaeudl, 2019], we show **(A)** the majority consensus tree (29 nodes and 28 edges), **(B)** the consensus network for *p* =20% (37 nodes and 41 edges), **(C)** the consensus network for *p* =5% (358 nodes and 843 edges), and **(D)** the consensus outline (106 nodes and 106 edges).

Given such a collection of phylogenetic trees, how to summarize them? One approach is to compute a consensus phylogentic tree, such as the strict-, majority-, or greedy consensus (Bryant, 2003), say. The strict-consensus tree contains only those branches (or, more precisely, splits) that are present in all input trees, where as the majority-consensus tree contains all branches that are present in more than half the input trees (see Figure 1A). The greedy-consensus tree is obtained by greedily determining a set of compatible splits so as to maximize the total support of the splits, where the support of a split is the number of input trees that contain it. Another approach is to provide a phylogenetic network to summarize the trees, such as a consensus network (Holland and Moulton, 2003). This is computed for a fixed threshold *p* by selecting all input splits that are present in at least *p* input trees and then constructing a split network to represent those splits (Dress and Huson, 2004) (see Figures 1B,C). An alternative is to provide a visual summary, such as a densi-tree visualization (Bouckaert, 2010), in which all trees are overlayed in a single drawing (see Figure 1D).

Commonly-used consensus trees can be efficiently computed and they aim at summarizing what the input trees have in common. One advantage of a consensus tree is that it uses *O*(*n*) nodes and edges to represent evolutionary relationships between *n* taxa. One drawback of such a consensus tree is that it provides a specific clustering of taxa with little indication of uncertainty or alternative groupings. This can be addressed, to some degree, by indicating the support of edges as the proportion of input trees that contain it, say.

Consensus networks operate by collecting all splits contained in more than a proportion of *p* input trees, and then using a split network (Huson and Bryant, 2006) to visualize the data. Such networks are able to display competing phylogenetic scenarios, that is, incompatible splits. Note that selecting $p=\frac{1}{2}$ will ensure that the set of collected splits is compatible and corresponds to the majority consensus tree (Bryant, 2003).

Smaller values may give rise to non-tree networks. In practice, choosing a suitable value of *p* can be challenging. If *p* is too large, then conflicting splits may not be represented. If *p* is too small, then the resulting set of splits may be “too incompatible” and require an exponential number of nodes and edges in the corresponding network. In practice, such networks can be complicated and difficult to comprehend.

Here we propose a new type of consensus calculation that we call a phylogenetic *consensus outline*. On the one hand, like a consensus tree, it can always be computed and visualized efficiently, whereas, on the other hand, like a consensus network, it is able to display incompatible phylogenetic scenarios. This consensus uses a phylogenetic outline for visualization (Bagci et al., 2021). This is essentially a split network (Bandelt and Dress, 1992) that uses only *O* (*n* ^2^) nodes and edges to represent a set of circular splits, as an outer-labeled planar graph.

To illustrate and compare the concepts of a consensus tree, consensus network and outline consensus, we use as input a set of 78 gene trees on 17 aquatic taxa, based on sequence alignments provided in (Gruenstaeudl, 2019) and computed as described further below. In Figure 1A we show the majority consensus tree, representing all splits present in more than *p* = 50% of all input trees. In Figures 1B,C we show the consensus networks displaying all splits present in *p* = 20% and *p* = 5% of all trees, respectively. In Figure 1D we show the consensus outline for the same set of trees. In all cases, edges are scaled to represent the number of trees that support them. While the consensus network for *p* = 5% (shown in Figure 1C) and the consensus outline (shown in Figure 1D) convey a similar visual impression of where the underlying gene trees disagree, the consensus network contains 358 nodes and 843 edges, while the consensus network contains only 106 nodes and 106 edges.

In the following, we first describe the algorithm. We will then will illustrate its usage and will compare it to the use of consensus trees, consensus networks and densi-trees. For this, we use two examples. The first is a Bayesian phylogenetic analysis of languages from New Guinea, using data downloaded from (Greenhill, 2015). The second is a multi-gene study of water lilies, based on multiple sequence alignments published in (Gruenstaeudl, 2019).

## 2 Methods

The majority consensus of a list of phylogenetic trees *H* is obtained by collecting the set of “majority splits” that are contained in more than half of the input trees and then computing the phylogenetic tree that represents this set of splits (Bryant, 2003). A phylogenetic consensus network (Holland and Moulton, 2003) is obtained by collecting all splits that are contained in a small fraction of all trees, $\frac{1}{3}$ say, and then computing the split network that represents those splits (Dress and Huson, 2004).

The key question in such approaches is which splits to keep? The majority splits have a straight-forward interpretation as “visualize what the majority of trees have in common”. However, the threshold used to compute a consensus network is not easy to interpret and is chosen by hand to balance the task of displaying incompatibilities in the dataset and avoiding too much visual complexity in the network.

The main idea in this paper is to compute a simple consensus network with the help of a *PQ*-tree (Booth and Luecker, 1976). For the purposes of this paper, a *PQ*-tree is a data structure that maintains the set of all linear orderings (of a set of taxa) that are compatible with a set of clusters that the tree has “accepted”. Here, to be compatible means that the elements of a cluster appear consecutively in the linear ordering. A *PQ*-tree is a rooted tree in which every internal node is either of type *P*, in which case its list of children is considered unordered, or of type *Q*, in which case its children are ordered. The leaves of the tree correspond to the given taxa. A *PQ*-tree accepts a cluster, if the tree can be updated so as to continue to be compatible with all previously accepted clusters, while also accommodating the presented cluster. A linear ordering can be extracted from the *PQ*-tree by a traversal of the tree taking into account that the children of a *P* node can be visited in any order, whereas the children of a *Q* node must visited in the given order, or in reverse.

Here is a brief outline of our algorithm. Setup an empty *PQ*-tree. Choose a fixed taxon. (Any taxon will do, the result will always be the same.) Extract all splits from the list of input trees and sort them by decreasing support, where the support of a split is the number of trees that contain it. Define the cluster associated with a split as the side of the split that does not contain the fixed taxon. If the *PQ*-tree accepts the cluster, then keep the split, otherwise discard it. The resulting set of splits is “circular”, by virtue of the fact that the set of associated clusters are compatible with a linear ordering, and thus can be represented by an order-labeled planar split network (Dress and Huson, 2004) or phylogenetic outline (Bagci et al., 2021).

In more detail, let *T* be a phylogenetic tree on taxon set *X*, with non-negative edge weights. We will use S(T) to denote the *split encoding* of *T*, that is, the set of all *splits* *S* = *A*∣*B*, consisting of two non-empty, non-overlapping subsets of *X* with *A* ∪ *B* = *X*, that arise by collecting the two sets of taxa that are separated by some edge *e* in *T*. For any such split, we will use *ω*(*S*) to denote the weight of *S*, which is given by the length of the corresponding edge *e*.

Choose some fixed taxon *x*_0_ ∈ *X* and then, for any split *S* on *X*, let $\bar{S}$ denote the split part that *does not* contain *x*_0_.

In the following, assume that our input consists of a collection *H* = {*T*_1_, *…* , *T*_*m*_} of *m* unrooted phylogenetic trees on a set of *n* taxa *X*. We compute a phylogenetic consensus outline for profile *H* using the following algorithm:

1) Determine the set $S_{H}=\cup_{T\in h}S\left(T_{i}\right)$ of all splits contained in any of the input trees, and define the *support* of a split to be the number of trees for which it occurs. Alternatively, if the edges of the input trees themselves carry support values, then the support of a split can also be defined as the sum of support values of the corresponding edges.
2) Let *P* be a *PQ*-tree (Booth and Luecker, 1976). For each split $S\in S_{H}$ , in order of decreasing support, attempt to insert $\bar{S}$ into *P*.
3) Let $S_{P}$ be the set of all splits *S* for which $\bar{S}$ was accepted by the *PQ*-tree *P*.
4) Extract an ordering *Z* from *P*.
5) Apply the phylogenetic outline algorithm to $S_{P}$ and *Z* to compute the visualization.

In the first three steps, we use a *PQ*-tree *P* to greedily collect a set of splits $S_{P}$ that are consistent with some linear ordering of the set of taxa *X*. This is based on the fact that a *PQ*-tree maintains the set of all linear orderings of *X* that are consistent with all clusters that have been successfully inserted. (We say that a cluster *C* is consistent with a linear ordering *Z*, if the elements of *C* appear consecutively in *Z*. Similarly, a split *S* is consistent with *Z*, if $\bar{S}$ is.) In the fourth step, we extract an ordering for $S_{P}$ , and then finally, in the fifth step we compute the outline.

The algorithm requires at most *O* (*mn* ^2^) steps. To see this, note that the number of input splits is *O* (*mn*) and sorting these by decreasing support can be done in *O* (*mn*) steps using radix sort. The *PQ*-tree algorithm performs an insert-if-possible operation in *O*(*n*) steps, and so the computation of $S_{P}$ requires *O* (*mn* ^2^) steps in total. An ordering can be extracted from *P* in *O* (*n* ^2^) steps and drawing the outline requires only *O* (*n* ^2^) steps (Bagci et al., 2021).

The consensus methods discussed here all aim at selecting an informative subset of the input splits. To provide a measure of how much information is retained, for each split, we determine what proportion of the total weight of each tree it provides, and then compare the sum of all such values for the output splits with the sum for all the input values, and report the ratio of sums as a percentage, with a value of 100% indicating that all input splits are also in the output. For the four graphs shown in Figure 1, the values are: a) 94.9%, b) 96.7%, c) 98.0% and d) 97.6%, suggesting that little information is lost when using a consensus outline rather than a consensus network.

## 3 Results

We have implemented the algorithm for computing a phylogenetic consensus outline in our open-source program SplitsTreeCE (https://github.com/husonlab/splitstree6). In this section we illustrate the application of the method and compare it to the use of the majority consensus tree, consensus networks and densi-tree visualizations.

### 3.1 Bayesian analysis and consensus outlines

TransNewGuinea.org is a database of New Guinea languages provided by Greenhill (2015). It currently contains 1,219 words from 34 language families and 14,257 cognate sets, obtained from 19 different sources. Each cognate set contains many words and each word is linked to various cognate sets, allowing the application of phylogenetic methods to the languages.

Greenhill (2015) demonstrates how Bayesian phylogenetic analysis can be used to analyze such data. We performed a similar analysis on a collection of cognate sets and languages that we downloaded from TransNewGuinea.org. For 13 languages of the Rigo area of the Central district of Papua, we downloaded 1,123 congate sets, and we coded these as binary data, based on the presence or absence of the cognate set in each language.

These 13 languages are categorized into two classes, Austronesian and Non-Austronesian, containing three and ten members, respectively (Dutton, 1970). Non-Austronesian languages are further divided into three catergories: The Kwalen languages (Humene, Uare and Mulaha), the Doromu languages (Bareika, Lafaika and Aramaika) and the Maria languages (Maranomu, Maiagolo, Uderi and Maria). Doromu and Maria form the Manubaran language family in a broader context and are more closely related to each other.

Bayesian analysis was performed using the BEAST2 framework (Bouckaert et al., 2019), employing a mutation-death model, which is suitable for binary data, using the settings described in (Greenhill, 2015). We ran the program for 30 million iterations, sampling every 1000-th iterations and discarding the first 300,000 iterations as burn-in.

**FIGURE 2:**
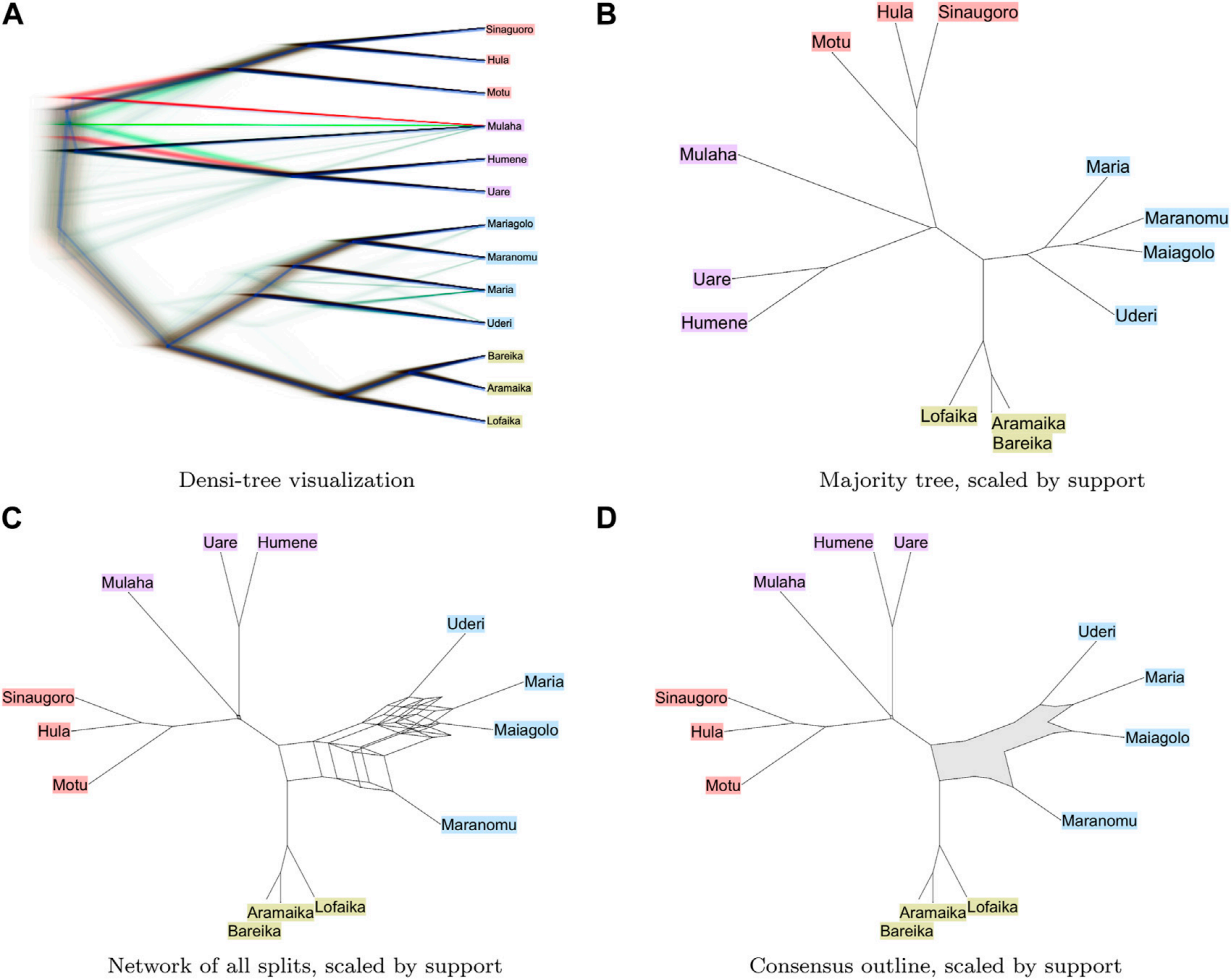
Bayesian phylogenetic analysis of languages from the Rigo area of the Central district of Papua. We show in **(A)** and **(B)**: a densi-tree visualization and the majority consensus tree respectively; **(C)** and **(D)**: a split network displaying all splits that are present in all trees and a consensus outline, respectively. We indicate Austronesian languages in red, Kwalen languages in purple, Doromu languages in green and Maria languages in blue [Dutton, 1970].

We summarize the sampled trees in Figure 2. Image a) was generated using the DensiTree program (Bouckaert, 2010). The images (b)–(d) were generating using SplitsTree (Huson and Bryant, 2006) (version 6.0.8-beta) and here edge lengths are scaled by the number of trees that have a corresponding edge. In all four visualizations, the two classes of languages are clearly separated. Among the Non-Austronesian languages, the placement of Maranomu appears to be the most conflicted. While it clusters closely with the other Maria languages (Maiagolo, Maria and Uderi), there is a some support for an alternative clustering with the Doromu languages (Aramika, Bareika and Lofaika). The alternative clustering is not obvious in the densi-tree visualization (Figure 2A) or in the majority tree (Figure 2B); however it is clearly suggested in a visualization of all splits contained in the input trees (Figure 2C) and the outline consensus (Figure 2D).

### 3.2 Multi-gene analysis and consensus outlines

Gruenstaeudl (2019) provides multiple sequence alignments for each of the 78 different protein coding genes that comprise the plastid genomes of 13 Nymphaeaceae (water lilies) and four other aquatic plants. For each of the alignments, we computed a maximum likelihood tree using RAxML v.8.2.12 (Stamatakis, 2014) with optimization of substitution rates and GAMMA model of rate heterogeneity. Branch supports were computed using 1000 bootstrap replicates using the rapid bootstrap analysis algorithm, obtaining 78 best-score gene trees, each on 17 taxa.

A main question addressed in (Gruenstaeudl, 2019) is whether the family of Nymphaeaceae is monophyletic, or whether the members of the *Numphar* genus prefer to cluster with the outgroup taxa, which are two members of the family of Cabombaceae and two members of the genus *Trithuria* in the family of Hydatellaceae.

**FIGURE 3:**
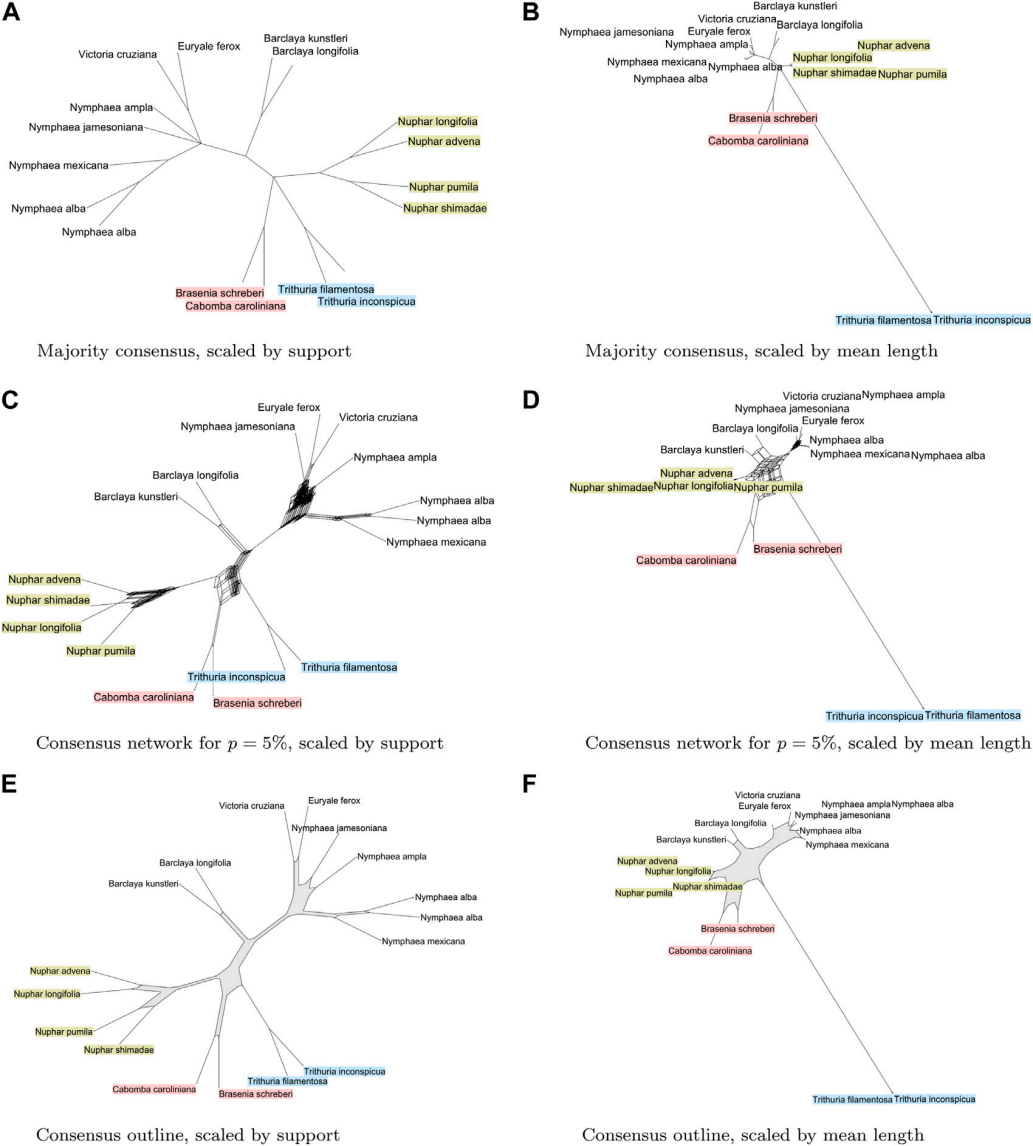
For 78 plasmid gene trees [Gruenstaeudl, 2019] from water lilies and other aquatic plants, we show **(A)** and **(B)**: the majority consensus tree, scaled by support, and by mean branch length, respectively; **(C)** and **(D)**: a split network displaying all splits that are present in >5% of all trees, scaled by support, and by mean branch length, respectively; and **(E)** and **(F)**: a consensus outline, scaled by support, and by mean branch length, respectively. We highlight *Trithuria* in blue, *Nuphar* in yellow and Cabombaceae in red.

Here we look into using different consensus calculations to shed light on this question. In the visualization of consensus splits, one can scale the length of edges to represent “support”, that is, the number of gene trees that contain the corresponding split. Another option is to scale by the mean branch length in the input trees, assuming that all trees have been scaled to the same total length. The majority consensus tree displays a polytomy between *Numphar*, Cabombaceae, *Trithuria*, and the set of nine remaining taxa (see Figures 3A, B). In contrast, a consensus network (using a threshold of 5%) indicates several different groupings of the genera in different combinations. The network contains higher-dimensional boxes that are very difficult to interpret (see Figures 3C, D). In contrast, the consensus outline clearly shows that the two members of the family Cabombaceae are grouped by some gene trees with the *Trithuria* genus, and are grouped by other gene trees with the *Nuphar* genus. Some gene trees support the placement of *Nuphar* taxa together with the Cabombaceae, where others place them closer to the other Nymphaeaceae (see Figures 3D, E).

## 4 Discussion

The motivation to use a phylogenetic network, rather than a phylogenetic tree, to represent the consensus of a collection of trees, is that a network has the potential to display incompatibilities among the input trees. The concept of a consensus network (Holland and Moulton, 2003) does this well when the data contains few incompatibilities, as shown in Figure 2. However, for more complex data, the consensus network requires a careful tuning of the threshold *p*; choosing *p* too close to $\frac{1}{2}$ may show no incompatibilities, whereas choosing smaller values of *p* can lead to an explosion of the number of nodes and edges required for visualization, as illustrated in Figures 3C,D.

Here we have introduced the concept of a phylogenetic consensus outline, which is computed by greedily collecting a circular set of splits from a collection of phylogenetic trees, which are then represented by a phylogenetic outline that contains only *O* (*n* ^2^) nodes and edges, where *n* is the number of taxa, as illustrated in Figures 3E,F. Our approach uses the classic *PQ*-tree algorithm (Booth and Luecker, 1976) and produces an network that is “outer-labeled planar”, thus ensuring that the displayed visualization is easy to grasp.

The outline consensus uses a greedy heuristic and is thus sensitive to the order in which choices are made. Slight differences in the input and differences in tie-breaking, might lead to very different solutions. Due to the *PQ*-tree acceptance criterion, on the one hand, it can happen that splits with strong support do not make it into the network. On the other hand, a split might make it into the final output, even if it is supported by only very few trees. To address the latter problem, our implementation allows edges to be scaled by support (number of trees that contain a given split) or by the sum of weights of all occurrences of the split in the input trees.

A related method, Neighbor-Net (Bryant and Moulton, 2004), uses a greedy approach to determine a circular ordering that is then used to compute a set of splits that give rise to a planar network, for a given input distance matrix. While Neighbor-Net suffers from the problems described above, in practice it is widely used. Neighbor-Net requires a distance matrix as input and thus could, in theory, be applied to a distance matrix computed from the input trees. An advantage of our method is that doesn’t require such a calculation and operates directly on the input splits.

One feature of Neighbor-Net is that it provides the user with a fit statistic indicating how well the network represents the input distances. In this paper we propose to report the proportion of total weight (normalized by tree lengths) of the output splits to indicate how well the network represents the input data.

Consensus outlines have a role to play between consensus trees and consensus networks. Like the former, consensus outlines provide clear graphical representations in the terms of an out-labeled planar graph. Like the latter, consensus outlines can visualize incompatible phylogenetic signals, but without burdening the user with higher-dimensional elements that are very difficult to interpret.

## Data Availability

The original contributions presented in the study are included in the article/Supplementary Material, further inquiries can be directed to the corresponding author.
